# Endoscopic mucosal resection versus endoscopic submucosal dissection for early colorectal cancer and precursor lesions: a retrospective study

**DOI:** 10.3389/fmed.2026.1849920

**Published:** 2026-07-01

**Authors:** Mengyuan Yang, Chanjuan Fan, Zhen Li, Liangqin Pan, Jianping Cheng

**Affiliations:** Department of Gastroenterology, Civil Aviation General Hospital, Beijing, China

**Keywords:** early colorectal cancer, endoscopic mucosal resection, endoscopic submucosal dissection, retrospective study, subgroup analysis

## Abstract

**Objective:**

Endoscopic mucosal resection (EMR) and endoscopic submucosal dissection (ESD) are both used for early colorectal cancer (CRC) and precursor lesions, but optimal size thresholds and subgroup-specific outcomes remain unclear. This study aimed to investigate the efficacy and safety of EMR versus ESD for early CRC and precursor lesions.

**Patients and methods:**

This single-center retrospective study enrolled 101 patients with early CRC or precursor lesions. EMR (*n* = 53) or ESD (*n* = 48) was selected based on lesion size, morphology, and suspected invasion depth. Pre-procedural evaluation included white-light endoscopy, chromoendoscopy, and endoscopic ultrasound/computed tomography for suspected deep invasion. Outcomes were operative time, en bloc, complete, and curative resection, and complications. Surveillance colonoscopy at 3, 6, and 12 months assessed recurrence at the resection site at the 12-month follow-up. Subgroup analyses included lesion diameter, location, pathology, and EMR resection type.

**Results:**

For lesions ≥20 mm, ESD achieved higher en bloc (91.2% vs. 71.4%, *p* = 0.036), complete (85.3% vs. 62.9%, *p* = 0.034), and curative (82.4% vs. 60.0%, *p* = 0.041) resection rates than EMR. Outcomes were similar for lesions <20 mm. Subgroup analysis showed a greater ESD benefit for lesions ≥30 mm. Right-colon ESD had a significantly higher complication rate than EMR (40.0% vs. 6.3%, *p* = 0.046). For high-grade dysplasia/cancer, en bloc resection was numerically higher with ESD (93.8% vs. 70.0%, *p* = 0.076). Complete resection was 40.0% with piecemeal EMR and 93.0% with en bloc EMR. Multivariable analysis identified ESD as an independent predictor of en bloc resection (OR 3.85, 95% CI 1.42–10.43, *p* = 0.008). At 12-month surveillance colonoscopy (median follow-up, 12.0 months), recurrence was 2.1% (1/48) for ESD and 11.3% (6/53) for EMR (*p* = 0.072).

**Conclusion:**

For lesions ≥20 mm, ESD provides superior resection outcomes, particularly for lesions ≥30 mm, but with higher complication rates, especially in the right colon. For lesions <20 mm, EMR is preferred. When en bloc EMR is not feasible, ESD should be prioritized over piecemeal EMR, particularly for lesions ≥30 mm.

## Introduction

Colorectal cancer (CRC), a disease driven by multiple etiological factors, is the third most commonly diagnosed malignancy and the second leading cause of cancer-related mortality worldwide (1, 2). Because early-stage CRC is often asymptomatic, timely detection and intervention are paramount to improving clinical outcomes (3). Endoscopic resection has become the standard of care for early CRC and its precursor lesions (4). Two principal techniques are used, including endoscopic mucosal resection (EMR), typically indicated for lesions smaller than 20 mm, and endoscopic submucosal dissection (ESD), which enables en bloc removal of larger or irregularly shaped lesions and provides intact pathological specimens (5). However, the colon’s narrow lumen, thin wall, and tortuous folds render ESD technically demanding, with longer procedure times and higher complication rates than EMR (6).

Previous comparisons of EMR and ESD have yielded conflicting results, largely due to small sample sizes, lack of detailed lesion-size stratification, and limited follow-up. A 2020 meta-analysis by Zhao et al. (7) demonstrated that ESD achieves higher en bloc and complete resection rates with lower recurrence compared with EMR for colorectal laterally spreading tumors. A 2022 systematic review from East Asian countries similarly reported that ESD leads to higher rates of en bloc and R0 resection, as well as lower rates of bleeding and recurrence, albeit with a higher risk of perforation (8). More recently, a meta-analysis (9) shows that ESD is more effective than EMR in achieving complete and curative resections for early esophageal cancer and squamous cell carcinoma, particularly for lesions >20 mm.

Despite these advances, several clinically relevant issues remain unresolved. The optimal size threshold for selecting ESD over EMR is still debated, and the influence of lesion location and pathological grade on outcomes has not been well characterized. Furthermore, the decision-making process for piecemeal versus en bloc EMR, and the role of ESD when en bloc EMR is not feasible, require further clarification. To fill these gaps, the present retrospective cohort study compared EMR and ESD for early CRC and precursor lesions. Key comparisons included operation time, en bloc resection, complete resection, curative resection, and complications stratified by lesion diameter. The impact of piecemeal versus en bloc EMR on resection quality was assessed, and short-term follow-up recurrence rates were reported. The findings are intended to provide evidence-based, size- and location-specific guidance for selecting the optimal endoscopic resection technique.

## Methods

### Study design and ethical approval

This single-center retrospective study followed the STROBE guidelines (10) and was conducted at Civil Aviation General Hospital, China. The study protocol was approved by the Institutional Ethics Board of the Civil Aviation General Hospital, Beijing, China (2022-L-K-45). The ethics committee waived the requirement for individual patient informed consent for this retrospective analysis because all clinical data were anonymized.

### Study participant

A total of 101 patients with early CRC and precursor lesions admitted to our hospital from January 2022 to January 2024 were enrolled and underwent either EMR (*n* = 53) or ESD (*n* = 48) based on clinical decision-making according to institutional protocols. Inclusion criteria were: (1) histopathological confirmation of early CRC or precursor lesions according to Chinese consensus criteria (11); (2) discontinuation of antiplatelet and anticoagulant agents for at least 1 week before the procedure. Patients were excluded if they had familial adenomatous polyposis, immune disorders, significant cardiopulmonary dysfunction, psychiatric disorders, concurrent invasive CRC or other malignancies, incomplete records or loss to follow-up, or withdrawal from the study.

### Pre-procedure evaluation

All patients underwent a standardized pre-procedure evaluation to assess lesion characteristics and guide the choice of resection technique. The evaluation began with high-definition white-light endoscopy to determine the lesion’s size, morphology, and location. Lesion morphology was classified according to the Paris endoscopic classification system (12): polypoid or non-polypoid. Laterally spreading tumors (LSTs) were further subclassified as granular or non-granular, with granular lesions subtyped as homogeneous or nodular mixed, and non-granular lesions as flat elevated or pseudodepressed. Subsequently, chromoendoscopy and narrow-band imaging with magnification were used to evaluate the mucosal pit pattern and microvascular architecture. Pit pattern was classified using the Kudo classification (13), and narrow-band imaging (NBI) findings were classified according to the Japan NBI Expert Team (JNET) classification (14). The presence of fibrosis was assessed based on the lifting sign after submucosal injection. For lesions exhibiting features suggestive of deep submucosal invasion, additional imaging was performed. Endoscopic ultrasound (EUS) was utilized to assess the depth of invasion and to evaluate for the presence of suspicious pericolorectal lymph nodes. Furthermore, for lesions with EUS features concerning for T1b cancer or deeper, or for lesions with suspected metastasis, an abdominopelvic computed tomography (CT) scan was obtained to rule out distant metastases. Lesions with suspected deep submucosal invasion were considered for ESD only if curative resection was deemed feasible, whereas lesions with evidence of deep invasion beyond endoscopic resectability were referred for surgical resection and not included in this study. During the pre-procedure evaluation, the endoscopist specifically assessed the feasibility of en bloc snare resection based on lesion size, morphology, location, and lifting characteristics. Lesions unsuitable for en bloc snare resection were considered for piecemeal EMR only if ESD was not available or was deemed higher risk due to lesion location or patient factors.

### Selection criteria for EMR versus ESD

The choice between EMR and ESD was made by the endoscopist based on established clinical protocols. Treatment allocation followed a systematic clinical algorithm incorporating lesion characteristics, imaging findings, and patient factors. The 20 mm threshold was based on established international guidelines, including the European Society of Gastrointestinal Endoscopy (ESGE) guidelines and the Japanese Gastroenterological Endoscopy Society guidelines, which recommend EMR for lesions <20 mm and ESD for lesions ≥20 mm (5, 15).

EMR was selected when: (1) lesion diameter <20 mm with a positive lifting sign and no endoscopic features suggesting deep submucosal invasion; (2) lesion diameter 20–29 mm with favorable morphology and the lesion could be completely ensnared in a single attempt; or (3) patient factors that increased the risk of ESD complications. EMR was preferred for lesions <20 mm in diameter with a positive lifting sign and no endoscopic features suggesting deep submucosal invasion. During EMR, en bloc resection was attempted for lesions with a maximum diameter ≤20 mm that demonstrated a positive lifting sign and favorable morphology. Piecemeal EMR was performed when en bloc snare resection was judged technically unfeasible, which occurred for lesions >20 mm in diameter with a transverse diameter exceeding the snare loop capacity, for lesions with irregular morphology that precluded complete ensnaring in a single attempt, or for lesions where the endoscopic view was suboptimal. In all cases of piecemeal EMR, the decision to proceed with piecemeal resection rather than ESD was documented before the procedure and was based on a combination of lesion characteristics and clinical judgment, including assessment of lesion location, patient comorbidities, and availability of ESD equipment and expertise.

ESD was selected when: (1) lesion diameter ≥30 mm, regardless of morphology; (2) lesion diameter 20–29 mm with features precluding en bloc snare resection; (3) lesions with suspected superficial submucosal invasion on EUS; (4) lesions where complete pathological assessment was deemed essential; or (5) recurrent lesions after previous endoscopic resection. For lesions in the 20–29 mm range, ESD was preferentially offered when the endoscopist judged that en bloc snare resection would be technically challenging or when complete pathological evaluation was considered clinically necessary. The decision to perform piecemeal rather than en bloc EMR was made by the endoscopist before the procedure, based on lesion size, morphology, and location. When a lesion was deemed unsuitable for en bloc EMR, ESD was preferentially offered as the alternative, provided that the lesion did not have deep submucosal invasion on pre-procedure evaluation. ESD was selected over piecemeal EMR for lesions ≥30 mm, for lesions with suspected superficial submucosal invasion, and for lesions where complete pathological assessment was deemed essential. The 30 mm threshold was chosen as a pre-specified exploratory cut-off to further characterize the relationship between lesion size and outcomes, based on emerging evidence suggesting that the benefit of ESD becomes more pronounced for larger lesions (16).

All endoscopists had performed at least 50 colorectal EMR and 50 colorectal ESD procedures before the study start to ensure competence in both techniques; thus, the choice of technique was driven primarily by lesion characteristics rather than operator inexperience. The final decision was documented before the procedure, and no patient was switched from one technique to the other intraoperatively.

### Procedures

#### ESD group

The operator selected an appropriate endoscope based on lesion characteristics and advanced it to the target area. The lesion was uniformly stained with 0.4% indigo carmine solution. NBI with magnification was used to assess tumor morphology and pit pattern, enabling precise delineation of the lesion margin. Marginal marking was performed with a mucosal incision knife at 3–5 mm from the lesion border, followed by submucosal injection. The lesion was then incised and dissected using the mucosal incision knife. Intraoperatively and postoperatively, hemostatic forceps were used to achieve hemostasis of bleeding points on the wound surface, margins, and exposed small vessels. Small perforations were closed with hemostatic clips.

#### EMR group

Submucosal injection was administered at the lesion base. A snare was introduced to encircle the lesion together with a margin of healthy surrounding mucosa. For lesions where the entire lesion could be captured within the snare loop, en bloc resection was performed using high-frequency electrosurgical current. Piecemeal resection was performed when the lesion could not be fully captured in a single snare attempt, either because of size, morphology, or technical factors. Piecemeal resection was accomplished by sequential snare resections, starting from the most accessible portion and proceeding systematically until the entire lesion was removed. High-frequency electrosurgical current was then applied to completely resect the entrapped lesion. The remainder of the procedure was identical to that of the ESD group.

### Outcome measures

Observation indicators included: (1) operation time, defined as the interval from submucosal injection to complete endoscopic lesion removal; (2) en bloc resection, defined as one-piece removal of the lesion yielding a continuous specimen; (3) complete resection defined as negative horizontal and vertical margins on histopathological examination; (4) curative resection, defined as complete lesion removal with submucosal invasion depth ≤1,000 μm and no lymphovascular invasion, poor differentiation, or high-grade tumor budding; and (5) complications, including intraprocedural and postprocedural bleeding, perforation, and post-electrocoagulation syndrome. For all resected specimens, pathological assessment included measurement of horizontal and vertical resection margins (mm) and, for lesions with invasive carcinoma, submucosal invasion depth (μm). Margins were considered positive if tumor cells were present at the inked margin. Submucosal invasion depth was measured from the muscularis mucosae to the deepest point of tumor invasion, using an ocular micrometer.

### Follow-up

All patients were scheduled for a standardized follow-up program after the index procedure: (1) a clinical assessment at 3 months post-procedure, including evaluation of symptoms and review of any adverse events; (2) a surveillance colonoscopy at 6 months post-procedure to assess the resection site for any visible residual or recurrent lesion, with biopsy of any suspicious mucosal changes; and (3) a second surveillance colonoscopy at 12 months post-procedure. Recurrence was defined as the presence of histologically confirmed neoplastic tissue at the original resection site during follow-up colonoscopy. Follow-up duration was calculated from the date of the index procedure to the date of the last surveillance colonoscopy or the date of last contact.

### Subgroup analyses

Prespecified subgroup analyses were performed by: (1) lesion diameter (using the guideline-based cut-off of 20 mm and a pre-specified exploratory cut-off of 30 mm); (2) location (right colon: cecum, ascending colon, hepatic flexure vs. left colon/rectum: transverse colon to rectum); (3) histopathological categories included low-grade intraepithelial neoplasia (LGD), high-grade intraepithelial neoplasia (HGD), and adenocarcinoma (AC); (4) within the EMR group including en bloc versus piecemeal resection; and (5) lesion morphological characteristics (Paris classification, LST subtype, pit pattern, NBI classification, and presence of fibrosis).

### Statistical analysis

Continuous variables are presented as mean ± standard deviation (SD) and were compared using independent *t*-tests after normality testing (Shapiro–Wilk). Categorical variables are expressed as frequencies (percentages) and were compared using χ^2^ or Fisher’s exact test. For non-normally distributed continuous variables (margin distance and invasion depth), data are presented as median with interquartile range (IQR) and compared using the Mann–Whitney U test. Multivariable logistic regression was performed for en bloc resection including lesion diameter, location, procedure, Paris morphology, LST subtype, and presence of fibrosis, and pathological category. All tests were two-tailed, and *p* < 0.05 was considered statistically significant. All subgroup analyses were pre-specified in the study protocol before data analysis. SPSS version 20.0 (IBM Corp., Armonk, NY, United States) was used.

## Results

### Baseline characteristics

A total of 101 patients were included. Baseline characteristics were similar between groups (Table 1). No significant differences were observed in age, sex, lesion location, or pathology (all *p* > 0.05). There were also no significant differences in lesion diameter between groups (mean 21.2 ± 8.5 mm for ESD vs. 19.8 ± 9.1 mm for EMR, *p* = 0.432), suggesting that lesion size alone did not strongly drive treatment allocation in this cohort.

**Table 1:** Baseline characteristics of patients.

Characteristic	ESD group (*n* = 48)	EMR group (*n* = 53)	*P*
Age, years, mean ± SD	59.05 ± 7.91	58.64 ± 7.89	0.795
Male, *n* (%)	28 (58.3)	32 (60.4)	0.835
Lesion location, *n* (%)			0.093
Sigmoid colon	26 (54.2)	20 (37.7)	
Rectum	17 (35.4)	17 (32.1)	
Ascending colon	3 (6.3)	11 (20.8)	
Other	2 (4.2)	5 (9.4)	
Lesion diameter, mm	21.2 ± 8.5	19.8 ± 9.1	0.432
Diameter categories, *n* (%)			0.452
<20 mm	14 (29.2)	18 (34.0)	
≥20 mm	34 (70.8)	35 (66.0)	
Pathology, *n* (%)			0.469
LGD	31 (64.6)	33 (62.3)	
HGD/AC	16 (33.3)	20 (37.7)	
Serrated adenoma	1 (2.1)	0 (0)	
Paris classification, *n* (%)			0.041
Ip/Isp	12 (25.0)	20 (37.7)	
Is	20 (41.7)	12 (22.6)	
IIa	10 (20.8)	14 (26.4)	
IIb/IIc	6 (12.5)	7 (13.2)	
LST subtype, *n* (%)			0.036
LST-G (homogeneous)	13 (27.1)	18 (34.0)	
LST-G (nodular mixed)	12 (25.0)	17 (32.1)	
LST-NG (flat elevated)	9 (18.8)	4 (7.5)	
LST-NG (pseudodepressed)	3 (6.3)	1 (1.9)	
Non-LST	11 (22.9)	13 (24.5)	
Kudo pit pattern, *n* (%)			0.039
I–II (normal/hyperplastic)	2 (4.2)	5 (9.4)	
III–IV (adenomatous)	32 (66.7)	38 (71.7)	
V (invasive)	7 (14.6)	2 (3.8)	
Not recorded	7 (14.6)	8 (15.1)	
JNET classification, *n* (%)			0.068
Type 1 (hyperplastic)	2 (4.2)	5 (9.4)	
Type 2A (low-grade dysplasia)	28 (58.3)	37 (69.8)	
Type 2B (high-grade dysplasia)	6 (12.5)	3 (5.7)	
Type 3 (deep invasive)	2 (4.2)	0	
Not recorded	10 (20.8)	8 (15.1)	
Fibrosis (poor/non-lifting), *n* (%)	9 (18.8)	4 (7.5)	0.092

EMR, endoscopic mucosal resection; ESD, endoscopic submucosal dissection; LGD, low-grade intraepithelial neoplasia; HGD, high-grade intraepithelial neoplasia; AC, adenocarcinoma; LST, laterally spreading tumor; LST-G, granular laterally spreading tumor; LST-NG, non-granular laterally spreading tumor; JNET, Japan NBI Expert Team.

Lesion characteristics, including Paris classification, LST subtype, pit pattern, NBI classification, and fibrosis, are shown in Table 1. Paris morphology distribution differed between groups, with the ESD group having higher proportions of Paris Is lesions (41.7% vs. 22.6%, *p* = 0.041) and LST-NG lesions (25.0% vs. 9.4%, *p* = 0.036). Kudo type V and JNET 2B/3 were more frequent in the ESD group (14.6% vs. 3.8%, *p* = 0.039; 16.7% vs. 5.7%, *p* = 0.068). Fibrosis was more frequent in the ESD group (18.8% vs. 7.5%, *p* = 0.092). LST-G subtype distribution and Kudo types I–IV did not differ significantly between groups (*p* = 0.572 and *p* = 0.184, respectively).

### Operation time and resection outcomes

Table 2 summarizes operation time and resection outcomes stratified by detailed lesion diameter categories. For lesions <20 mm, operation time was significantly shorter with EMR (mean difference 26.6 min, 95% CI 19.8–33.4, *p* < 0.001), but resection outcomes did not differ. For lesions 20–29 mm, operation time remained shorter with EMR (*p* < 0.001), whereas en bloc, complete, and curative resection rates were numerically but not significantly higher with ESD. For lesions 30–39 mm and ≥40 mm, ESD consistently achieved higher resection rates, although differences did not reach statistical significance owing to small subgroup sample sizes. For lesions ≥20 mm overall, ESD had significantly higher en bloc (91.2% vs. 71.4%, *p* = 0.036), complete (85.3% vs. 62.9%, *p* = 0.034), and curative (82.4% vs. 60.0%, *p* = 0.041) resection rates.

**Table 2:** Operation time and resection outcomes by lesion diameter.

Lesion diameter	Group	*n*	Operation time (min)	En bloc resection	Complete resection	Curative resection
<20 mm	ESD	14	48.37 ± 7.32	14 (100%)	13 (92.9%)	12 (85.7%)
	EMR	18	21.77 ± 3.59	17 (94.4%)	16 (88.9%)	15 (83.3%)
	*P*		<0.001	0.898	0.819	0.759
20–29 mm	ESD	18	58.6 ± 8.3	17 (94.4%)	16 (88.9%)	15 (83.3%)
	EMR	20	31.2 ± 4.8	16 (80.0%)	14 (70.0%)	13 (65.0%)
	*P*		<0.001	0.195	0.162	0.204
30–39 mm	ESD	10	78.4 ± 10.1	9 (90.0%)	8 (80.0%)	8 (80.0%)
	EMR	9	42.5 ± 5.9	6 (66.7%)	5 (55.6%)	5 (55.6%)
	*P*		<0.001	0.204	0.239	0.239
≥40 mm	ESD	6	95.2 ± 12.5	5 (83.3%)	5 (83.3%)	5 (83.3%)
	EMR	6	51.3 ± 7.2	3 (50.0%)	3 (50.0%)	3 (50.0%)
	*P*		<0.001	0.272	0.272	0.272

Median horizontal margin distance was 2.5 mm (IQR, 1.8–3.5) for ESD and 2.0 mm (IQR, 1.5–3.0) for EMR (*p* = 0.084); median vertical margin distance was 1.8 mm (IQR, 1.2–2.5) and 1.5 mm (IQR, 1.0–2.2), respectively (*p* = 0.112). Among 36 patients with invasive adenocarcinoma (16 ESD, 20 EMR), median submucosal invasion depth was 650 μm (IQR, 400–850) and 720 μm (IQR, 480–920), respectively (*p* = 0.361). All patients with invasion depth ≤1,000 μm and negative margins met curative resection criteria.

### Complications and follow-up outcomes

Complications and follow-up outcomes are summarized in Table 3. The overall complication rate was significantly higher in the ESD group compared with the EMR group (18.8% vs. 5.7%, *p* = 0.042). All intraoperative perforations in the ESD group were successfully closed with clips, and none required emergency surgery; no procedure-related deaths occurred. The median follow-up duration was 12.0 months (IQR: 10.0–14.0 months), with no significant difference between the ESD and EMR groups (*p* = 0.382). At the 12-month follow-up, surveillance colonoscopy was completed in 98 of 101 patients (97.0%). Local recurrence was detected in 7 patients: 1 of 48 (2.1%) in the ESD group and 6 of 53 (11.3%) in the EMR group (*p* = 0.072).

**Table 3:** Complications and follow-up of patients.

Outcomes	ESD group (*n* = 48)	EMR group (*n* = 53)	*P*
Complication			
Intraoperative bleeding	2 (4.2%)	2 (3.8%)	
Intraoperative perforation	2 (4.2%)	0	
Delayed bleeding	2 (4.2%)	1 (1.9%)	
Post-electrocoagulation syndrome	3 (6.3%)	0	
Total	9 (18.8%)	3 (5.7%)	0.042
Follow-up			
Median follow-up, months (IQR)	12.5 (10.0–15.0)	12.0 (10.0–14.0)	0.382
12-month surveillance colonoscopy, *n* (%)	47 (97.9%)	51 (96.2%)	0.613
Recurrence at 12 months, *n* (%)	1 (2.1%)	6 (11.3%)	0.072

EMR, endoscopic mucosal resection; ESD, endoscopic submucosal dissection.

### Subgroup analyses

Table 4 presents subgroup analyses by lesion location, pathological category, EMR resection type, and morphological characteristics. Right-colon ESD had a significantly higher complication rate than right-colon EMR (40.0% vs. 6.3%, *p* = 0.046), with no significant difference in en bloc resection. In the left colon/rectum, ESD achieved a significantly higher en bloc resection rate (95.3% vs. 81.1%, *p* = 0.044) without a significant increase in complications. In HGD/AC lesions, ESD showed numerically higher en bloc (93.8% vs. 70.0%, *p* = 0.076) and complete (87.5% vs. 60.0%, *p* = 0.065) resection rates, approaching statistical significance. In LGD lesions, no significant differences were observed. Piecemeal EMR (*n* = 10, all lesions ≥20 mm) had a significantly lower complete resection rate than en bloc EMR (40.0% vs. 93.0%, *p* < 0.001).

**Table 4:** Subgroup analyses for outcomes by location, pathology, and EMR resection type.

Subgroup	Outcome	ESD group	EMR group	*P*
Location: Right colon	En bloc resection	4/5 (80.0%)	12/16 (75.0%)	0.814
	Complication rate	2/5 (40.0%)	1/16 (6.3%)	0.046
Location: Left colon/rectum	En bloc resection	41/43 (95.3%)	30/37 (81.1%)	0.044
	Complication rate	7/43 (16.3%)	2/37 (5.4%)	0.121
Pathology: HGD/AC	En bloc resection	15/16 (93.8%)	14/20 (70.0%)	0.076
	Complete resection	14/16 (87.5%)	12/20 (60.0%)	0.065
Pathology: LGD	En bloc resection	29/31 (93.5%)	28/33 (84.8%)	0.276
	Complete resection	28/31 (90.3%)	26/33 (78.8%)	0.203
EMR type: En bloc	Complete resection	–	40/43 (93.0%)	–
EMR type: Piecemeal	Complete resection	–	4/10 (40.0%)	–
Paris Is morphology	En bloc resection	18/20 (90.0%)	8/12 (66.7%)	0.038
LST-G	En bloc resection	16/17 (94.1%)	19/22 (86.4%)	0.317
LST-NG	En bloc resection	11/12 (91.7%)	3/5 (60.0%)	0.045
Fibrosis present	En bloc resection	8/9 (88.9%)	2/4 (50.0%)	0.043
No fibrosis	En bloc resection	36/39 (92.3%)	36/41 (87.8%)	0.314

EMR, endoscopic mucosal resection; ESD, endoscopic submucosal dissection; LGD, low-grade intraepithelial neoplasia; HGD, high-grade intraepithelial neoplasia; AC, adenocarcinoma; LST-G, granular laterally spreading tumor; LST-NG, non-granular laterally spreading tumor.

By Paris classification, ESD achieved higher en bloc resection rates than EMR for all subtypes, most pronounced for Is lesions (ESD 90.0% vs. EMR 66.7%; *p* = 0.038). Rates were comparable for LST-G (94.1% vs. 86.4%; *p* = 0.317), whereas for LST-NG, ESD yielded a significantly higher rate (91.7% vs. 60.0%; *p* = 0.045). In fibrotic lesions, ESD achieved a higher en bloc rate (88.9% vs. 50.0%; *p* = 0.043); in non-fibrotic lesions, the difference was non-significant (92.3% vs. 87.8%; *p* = 0.314).

### Multivariable analysis

Multivariable logistic regression for en bloc resection (Table 5) identified ESD as an independent positive predictor (OR 3.85, 95% CI 1.42–10.43, *p* = 0.008) and larger lesion diameter as a negative predictor (OR 0.89, 95% CI 0.82–0.96, *p* = 0.003). Right-colon location and HGD/AC pathology showed negative trends but were not statistically significant.

**Table 5:** Multivariable logistic regression for en bloc resection.

Variable	Odds ratio	95% CI	*P*
Lesion diameter (per 1 mm increase)	0.89	0.82–0.96	0.003
Right colon (vs other locations)	0.42	0.15–1.18	0.099
ESD (vs EMR)	3.85	1.42–10.43	0.008
HGD/AC (vs LGD)	0.58	0.22–1.52	0.267

EMR, endoscopic mucosal resection; ESD, endoscopic submucosal dissection; LGD, low-grade intraepithelial neoplasia; HGD, high-grade intraepithelial neoplasia; AC, adenocarcinoma; CI, confidence interval.

## Discussion

In this retrospective comparative study of 101 patients with early CRC or precursor lesions, we found that ESD was superior to EMR for lesions ≥20 mm in terms of en bloc, complete, and curative resection rates, while EMR offered comparable efficacy with shorter operation time and lower complication rates for lesions <20 mm. Our subgroup analyses refined this recommendation by showing that ESD’s advantage becomes more pronounced for lesions ≥30 mm, that right-colon ESD carries a significantly higher complication risk, and that piecemeal EMR is associated with poor complete resection and higher recurrence. The follow-up further demonstrated that recurrence at the 12-month surveillance colonoscopy was substantially higher in the EMR group.

A meta-analysis (9) of randomized controlled trials reported that for colorectal polyps ≥20 mm, ESD significantly reduced recurrence compared with EMR. Our en bloc resection rates are consistent with pooled estimates from a previous meta-analysis, and operation times are comparable to those reported in Asian cohorts (7, 17). The detailed diameter subgroup analysis revealed that the superiority of ESD was not uniform across all larger lesions. For lesions 20–29 mm, ESD did not significantly outperform EMR, whereas for lesions ≥30 mm, the difference became more evident. This finding supports the selection of 30 mm as an exploratory threshold to better capture the size-dependent treatment effect, as the benefit of ESD appears to increase with lesion size. This suggests that the optimal size threshold may be higher than 20 mm, consistent with a large cohort study that identified lesion diameter ≥40 mm as an independent risk factor for recurrence after ESD (16).

ESD was preferentially used for lesions with more complex morphological features, including Paris Is lesions, LST-NG subtype, and lesions with fibrotic changes. This reflects appropriate clinical judgment, as these features are known to increase the technical difficulty of EMR and the risk of incomplete resection (18). The subgroup analysis by morphological characteristics confirmed that ESD’s advantage was most pronounced in these complex lesions: for LST-NG lesions, ESD achieved en bloc resection in 91.7% compared with 60.0% for EMR (*p* = 0.045), and in lesions with fibrosis, ESD achieved 88.9% versus 50.0% for EMR (*p* = 0.043). These findings are consistent with previous studies demonstrating that LST-NG morphology and fibrosis are independent predictors of difficult EMR and lower en bloc resection rates (19). Conversely, for LST-G lesions and those without fibrosis, where EMR en bloc resection rates were acceptable, the incremental benefit of ESD was less pronounced.

Within the EMR group, piecemeal resection was associated with a strikingly low complete resection rate (40.0% vs. 93.0% for en bloc EMR, *p* < 0.001) and accounted for all recurrences in the EMR arm, which aligns with a large cohort study (15). In our cohort, piecemeal EMR was reserved for lesions ≥20 mm in which en bloc snare resection was judged technically unfeasible, either due to size, morphology, or anatomical constraints. The poor outcomes associated with piecemeal EMR support the recommendation that ESD should be the preferred approach when en bloc EMR is not feasible. This is consistent with the ESGE clinical guidelines, which recommend ESD for lesions that cannot be resected en bloc by EMR, particularly when complete pathological assessment is required (15). Our data suggest that the threshold for switching from EMR to ESD should be guided not solely by lesion size but also by the anticipated feasibility of en bloc resection. For lesions ≥30 mm or for lesions with morphological features that preclude en bloc snare resection, ESD should be strongly considered over piecemeal EMR.

The location-based subgroup analysis provides important safety insights. Right-colon ESD had a complication rate of 40.0% versus 6.3% for EMR (*p* = 0.046). This is consistent with a retrospective study that reported lower en bloc and success rates in the right colon and a trend toward higher complications (20). An international multicenter study found right-colon location to be an independent risk factor for delayed bleeding after ESD, and prophylactic clip closure significantly reduced bleeding risk (21). Our findings reinforce that right-colon ESD should be performed with caution, and when indicated, prophylactic closure of the post-ESD defect should be strongly considered. The pathology subgroup analysis showed that in HGD/AC lesions, ESD achieved numerically higher en bloc (93.8% vs. 70.0%, *p* = 0.076) and complete (87.5% vs. 60.0%, *p* = 0.065) resection rates, approaching statistical significance despite the small sample size. This is clinically meaningful because incomplete resection of high-grade dysplasia may lead to rapid progression. A meta-analysis reported that HGD is a risk factor for recurrence after EMR (22). Therefore, for lesions with suspected HGD or early cancer, ESD should be the preferred technique to achieve R0 resection and minimize residual disease.

It is important to acknowledge that several modified EMR variants have emerged in recent years. Underwater EMR (U-EMR), which involves performing snare resection without submucosal injection while the colonic lumen is filled with water, has shown promising results for large colorectal lesions compared with conventional EMR (23, 24). Simplified EMR (s-EMR), which uses a dedicated cap-fitted device, has been proposed as an alternative for lesions in challenging locations (25). Cold EMR, performed without electrocautery, offers a favorable safety profile for diminutive polyps (26). Pre-cutting EMR has been reported to achieve higher en bloc resection rates for lesions 20–30 mm compared with standard EMR (27). Future studies directly comparing these modified EMR techniques with ESD will be valuable to determine whether they can achieve ESD-like outcomes with lower cost and complexity. Beyond these established and modified EMR techniques, even more advanced endoscopic resection methods have emerged in recent years, particularly for the management of rectal neuroendocrine tumors (rNETs). Endoscopic intermuscular dissection (EID) is a novel technique that enables removal of the mucosa, submucosa, and inner circular muscle layer while preserving the integrity of the rectal wall. EID has garnered attention for its efficacy in reducing positive vertical margins by allowing deeper dissection than conventional ESD. A retrospective study by Han et al. (28) comparing EID and ESD for rNETs found that both achieved high histological complete resection rates (92.3% vs. 100%, *p* > 0.99), although EID required longer procedure time (38.31 vs. 29.54 min, *p* = 0.036). EID may be a feasible option for selected lesions with suspected deep submucosal invasion requiring adequate vertical margins.

The present study did not include a formal cost analysis, which represents an important limitation in translating our clinical findings into practice. ESD generally entails higher procedural costs than EMR due to the requirement for specialized devices (e.g., electrosurgical knives, hemostatic forceps, injection needles), longer procedure times, and greater consumable utilization (27, 29, 30). The higher cost must be balanced against the potential benefits: reduced recurrence rates, fewer repeat procedures, and superior histopathological assessment. For lesions <20 mm, where outcomes are comparable, the cost-effectiveness clearly favors EMR. For lesions ≥20 mm, particularly those ≥30 mm, the higher upfront cost of ESD may be justified by the reduction in recurrence and the avoidance of piecemeal resection-related complications. However, formal cost-effectiveness analyses incorporating long-term surveillance and recurrence management are needed to establish the optimal economic threshold. Our findings provide the clinical efficacy data necessary to inform such analyses, but we caution against indiscriminate ESD use without considering local resource availability and cost implications.

Several limitations must be acknowledged. First, the retrospective, single-center design introduces potential selection bias. Although we have detailed the clinical criteria that guided the choice of EMR versus ESD, and despite multivariable adjustment for measured confounders, unmeasured factors such as subtle lesion morphology or endoscopist preference may still have influenced treatment assignment and outcomes. Second, while we have described a standardized pre-procedure evaluation protocol that included EUS and CT for suspicious lesions, the retrospective nature of the study precluded a systematic quantitative analysis of the impact of these imaging findings on the final outcomes. Third, the small sample size, particularly in subgroup analyses, limits statistical power and precision of estimates. Consequently, the nonsignificant differences observed in these subgroups should not be interpreted as evidence of equivalence, and the observed trends should be considered hypothesis-generating rather than confirmatory. The small sample sizes also mean that the statistically significant finding of higher complication rates with right-colon ESD, while noteworthy, is based on a very limited number of patients and requires confirmation in larger cohorts. Fourth, the median follow-up of 12 months is relatively short, and longer follow-up is needed to capture late recurrences; however, our 12-month surveillance data provide a robust assessment of early recurrence. Fifth, we did not systematically assess submucosal fibrosis, a known predictor of difficult ESD and complications. Lastly, the study was conducted at a tertiary center with experienced endoscopists, limiting generalizability to lower-volume centers.

## Conclusion

For early CRC and precursor lesions ≥20 mm, ESD provides superior en bloc, complete, and curative resection rates compared with EMR, with the advantage becoming more pronounced for lesions ≥30 mm. However, ESD carries a higher complication rate, especially in the right colon. For lesions <20 mm, EMR offers comparable efficacy with shorter operation time and lower complications. Piecemeal EMR should be avoided when en bloc resection is not feasible; ESD is preferred for such cases, particularly for lesions ≥30 mm. The higher recurrence rate observed with piecemeal EMR at 12-month follow-up further supports this recommendation. These clinical advantages must be considered alongside the higher procedural costs of ESD, which may limit its widespread adoption in resource-constrained settings.

## Data Availability

The original contributions presented in the study are included in the article/supplementary material, further inquiries can be directed to the corresponding author.
